# Antimicrobial Susceptibility Patterns of Salmonella Species in Southern Pakistan

**DOI:** 10.7759/cureus.4379

**Published:** 2019-04-03

**Authors:** Ghulam Shabbir Laghari, Zahid Hussain, Syed Zohaib Maroof Hussain, Haresh Kumar, Syed Mohammad Mazhar Uddin, Aatera Haq

**Affiliations:** 1 Pediatrics, Liaquat University of Medical and Health Sciences, Jamshoro, PAK; 2 Pediatrics, National Institute of Child Health, Karachi, PAK; 3 Surgery, Aga Khan University Hospital, Karachi, PAK; 4 Internal Medicine, United Medical and Dental College, Karachi, PAK; 5 Internal Medicine, Civil Hospital, Karachi, PAK; 6 Surgery, Civil Hospital, Karachi, PAK

**Keywords:** antibiogram, salmonella typhoid, antibiotic resistance, enteric fever, mdr typhoid, xdr typhoid, south pakistan

## Abstract

Introduction

Typhoid fever is a major infectious disease among the pediatric population of Pakistan. With inappropriate use of antibiotics and rising trends of multidrug-resistant (MDR) and extended drug-resistant (XDR) typhoid, it is becoming a public health emergency. This study evaluated the current trends in antibiotic susceptibilities to Salmonella (S) typhi and paratyphi A, B, and C in southern Pakistan.

Materials and methods

This cross-sectional study, conducted in the Pediatrics Department, Civil Hospital, Jamshoro from July to December 2018, included children with S. typhi and S. paratyphi A and B strains isolated from the laboratory-based culture of blood samples.

Results

There were 223 (81.1%) children with S. typhi and 52 (18.9%) with S. paratyphi isolates. Their mean age was 5 ± 3 years. The most common age group with S. typhi strains was two to five years (n = 102; 37.1%). Previous trials of antibiotics were taken by 162 (58.9%) children; 65 (40.1%) of these were physician-prescribed. Cefixime was most commonly taken (66.6%), followed by ciprofloxacin (33.3%). Cefixime and ceftriaxone showed 60.9% and 65.8% sensitivity, respectively. Ciprofloxacin sensitivity was seen in 50.1% S. typhi isolates. There were six (2.6%) cases of MDR typhoid and two (0.9%) cases of XDR typhoid.

Conclusion

Resistance to second-line antityphoid agents is increasing. Therefore, there is a need to modify prescribing behavior. The outbreak of XDR typhoid among children is an alarming public health concern for Pakistan. Widespread antibiotic stewardship programs must be conducted.

## Introduction

The two rod-shaped, gram-negative bacilli Salmonella enterica serovar typhi (S. typhi) and paratyphi (S. paratyphi) A, B, and C cause typhoid and paratyphoid enteric infections, respectively. The only reservoir for S. typhi is humans; its transmission is via fecal-oral route [1]. According to the Global Health Data Exchange, approximately 116,800 deaths were reported globally due to typhoid fever in 2017. Of these, there were about 79,000 deaths in South Asia, with 6,700 being reported from Pakistan [2].

The microbe is transmitted when food or water contaminated with human feces is consumed. Populations under risk include those without access to safe drinking water and those living in poor sanitation conditions and inadequate sewerage management [3]. In a literature review published by Mogasale et al., the odds of developing typhoid fever among those who have consumed unsafe water were 1.06 to 9.26 [4]. Poor and overcrowded communities and children of a younger age are also at higher risk [3]. In a population-based prospective surveillance study from five Asian countries, 57% of all S. typhi isolated from blood culture were from children of age 5 to 15 years. Typhoid incidence per annum in this age group was 412.9 per 100,000 persons in Pakistan and 493.5 per 100,000 persons in India [5].

The pattern of prescriptions in the management of enteric fever has also evolved. The most salient factor driving a change in prescription patterns is the emergence of drug resistance. Traditional first-line drugs (e.g., chloramphenicol, ampicillin, and trimethoprim-sulfamethoxazole) have failed to provide sufficient eradication since the late 1990s due to multidrug-resistant (MDR) typhoid [5,6]. The incidence of MDR typhoid has been reported to be 17% to 23% in South Asia [6,7]. The trend shifted from first-line antityphoidal drugs to first- and third-generation cephalosporins and fluoroquinolones (FQ) (second-line agents for typhoid), and azithromycin [6,8]. Concurrently, newer studies have started to report increasing antimicrobial resistance to one or more second-line antityphoid agents [8-10]. The factors contributing to the evolution of antibiotic resistance include monotherapy, incomplete antibiotic use directions, discontinuation of antibiotic therapy by the patients, and prescriptions without biochemical testing [8]. Finally, in 2016, the first case of extended drug-resistant (XDR) typhoid was reported from Hyderabad, a small city in the South of Pakistan. XDR typhoid is defined as the isolate resistant to all first-line antityphoid agents, ceftriaxone (CFX), and FQ [11]. Since then, the World Health Organization has reported more than 5,200 cases of XDR typhoid from Pakistan; all of which were from southern Pakistan [12].

The present study was conducted to evaluate the current trends in antibiotic susceptibilities to S. typhi and S. paratyphi A, B, and C isolates from blood cultures in the pediatric population of southern Pakistan.

## Materials and methods

A cross-sectional study was conducted at the Department of Pediatrics, Civil Hospital, Jamshoro in collaboration with the hospital laboratory. From July to December 2018, all children with S. typhi and S. paratyphi A and B strains isolated from the laboratory-based culture of blood samples were included in the study, after informed consent was obtained. The children were of age 18 or younger. All blood samples were of venous origin and collected by sterile measures.

For each child, 5 mL of venous blood was drawn and inoculated in special blood culture bottles containing 30 mL brain heart infusion (BHI) broth (Oxoid, England) and were incubated for five to seven days at 35 ± 2°C. BHI was used as a culture medium as it ensures the growth of all common microbes that can cause bacterial infections. Blood samples that were positive for S. typhi or S. paratyphi were subcultured on McConkey agar (Oxoid, England) for another two days at 35 ± 2°C as per standard methods. API-20E (Biomerieux, France), the analytical profile index system specific for differentiating between members of Gram-negative Enterobacteriaceae, was utilized for biochemical testing [13]. Serotyping was done by group and type-specific antisera (Bio-Rad). Kirby-Bauer disc diffusion technique on Mueller Hinton agar (Oxoid, England) was utilized to determine the patterns of antimicrobial sensitivity [14]. The agar plated were incubated aerobically, at 35 ± 2°C, for 24 hours. The antimicrobial discs utilized in this study are shown in Table 1.

**Table 1:** Antibiotic disc size on Kirby-Bauer disc diffusion method.

Antibiotic	Zone of inhibition on Kirby-Bauer disc diffusion test
Ampicillin	10 µg
Azithromycin	15 µg
Cefixime	5 µg
Cefotaxime	30 µg
Ceftriaxone	30 µg
Chloramphenicol	30 µg
Cotrimoxazole	1.25/23.75 µg
Ciprofloxacin	5 µg
Enoxacin	5 µg
Ertapenem	5 µg
Imipenem	10 µg
Meropenem	10 µg
Ofloxacin	5 µg

The data were entered and analyzed using IBM SPSS Statistics for Windows, Version 22.0 (IBM Corp., Armonk, NY). Frequencies and percentages were calculated.

## Results

There were 275 strains of S. typhi and S. paratyphi isolated during the study duration. There were 223 (81.1%) S. typhi isolates and 52 (18.9%) S. paratyphi isolates. There were 163 (59.3%) boys and 112 (40.7%) girls. The mean age of these children was 5 ± 3 years. The most common age group was two to five years (n = 102; 37.1%). The frequency and percentages of S. typhi and S. paratyphi strain according to the age and gender of the children are shown in Table 2.

**Table 2:** Age and gender distribution of the isolated species of salmonellae.

Patient Characteristics	S. typhi n (%)	S. paratyphi A/B n (%)	Total n (%)
Age			
<2 years	42 (18.8%)	21 (40.3%)	63 (22.9%)
2–5 years	86 (38.5%)	16 (30.7%)	102 (37.1%)
5–10 years	30 (13.4%)	12 (23.0%)	42 (15.3%)
11–18 years	65 (29.1%)	3 (5.7%)	68 (24.7%)
Gender			
Male	124 (55.6%)	39 (75%)	163 (59.3%)
Female	99 (44.4%)	13 (25%)	112 (40.7%)

The most frequent presenting concern among the study sample was high-grade fever with abdominal pain and/or vomiting (n = 173; 62.9%). Characteristic rose spots were only seen in 45 children (16.3%). There were 93 (33.8%) samples that were isolated from admitted children (all were S. typhi isolates), and the remaining 182 (66.2%) were seen in the outpatient department. Of the admitted children, 17 (18.2%) cases were complicated enough to require intensive care; nine (9.6%) complicated into disseminated intravascular coagulation (DIC) and one (1%) child complicated into intestinal obstruction which was surgically managed.

A previous trial of antibiotics was taken by 162 (58.9%) children; 65 (40.1%) of these were prescribed by a physician, and 97 (59.8%) were taken without any doctor's advice. The most common antibiotics taken were cefixime by 108 (66.6%) children followed by ciprofloxacin taken by 54 (33.3%) children.

The antimicrobial sensitivity patterns of S. typhi and S. paratyphi strains are shown in Table 3.

**Table 3:** Antibiotic sensitivity trend of S. typhi (n = 223) and S. paratyphi (n = 52) strains.

Antibiotic Agent	S. typhi n (%)	S. paratyphi A/B n (%)
Ampicillin	182 (81.6%)	52 (100%)
Azithromycin	211 (94.6%)	52 (100%)
Cefixime	136 (60.9%)	40 (76.9%)
Cefotaxime	201 (90.1%)	49 (94.2%)
Ceftriaxone	181 (65.8%)	50 (96.1%)
Chloramphenicol	215 (96.4%)	50 (96.1%)
Cotrimoxazole	215 (96.4%)	52 (100%)
Ciprofloxacin	138 (50.1%)	35 (67.3%)
Enoxacin	201 (90.1%)	52 (100%)
Ertapenem	222 (99.5%)	52 (100%)
Imipenem	196 (87.8%)	50 (96.1%)
Meropenem	203 (91.0%)	50 (96.1%)
Ofloxacin	220 (98.6%)	48 (92.3%)

S. typhi strains showed the highest sensitivity to ertapenem and ofloxacin; the lowest sensitivity seen was to cefixime. The higher sensitivity to chloramphenicol and cotrimoxazole indicated that the trend of resistance to first-line agents may be reversing. Bacterial sensitivity to cefixime and CFX was 60.9% and 65.8%, respectively, which is alarming since these are commonly used empirical agents against salmonella. Only half of the S. typhi isolates showed sensitivity to ciprofloxacin (50.1%) which is also a rising concern. Complete sensitivity to ampicillin, azithromycin, enoxacin, and ertapenem was seen in S. paratyphi strains. In the S. typhi group, there were six (2.6%) cases of MDR typhoid and two (0.9%) cases of XDR typhoid as shown in Table 4. Both cases of XDR typhoid complicated into DIC and required intensive care. One of these children was sensitive to meropenem and azithromycin, and the other was sensitive to meropenem and imipenem. Both children recovered over time.

**Table 4:** Incidence of multi-drug and extended-drug resistance to S. typhi strains. MDR: Multidrug-resistant; XDR: Extended drug-resistant.

Drug Resistant Strains	S. typhi n (%)
MDR strains (resistant to 1^st^ line agents – ampicillin, cotrimoxazole, and chloramphenicol)	6 (2.6%)
XDR strains (resistant to 1^st^ line agents + ceftriaxone + fluoroquinolone)	2 (0.9%)

## Discussion

The observed incidence of typhoid was higher in pre-school children of age two to five years and among male children. One-third of the children needed hospital admission, and almost 60% had previously taken antibiotics for fever; more than half of these were not prescribed by a physician. The rising incidence of XDR typhoid in southern Pakistan is alarming and requires immediate attention. The trends of antimicrobial sensitivity are evolving. In the pediatric population, this study reports six cases of MDR and two cases of XDR. However, the sensitivity to oral and injectable second- and third-generation cephalosporins is low; the case is similar for ciprofloxacin. However, higher sensitivity to azithromycin and ampicillin was seen which indicates that prescription patterns for empirical, outpatient management of typhoid might be shifted towards these agents. Injectable antibiotics including carbapenems must be saved for complicated cases.

In another local study, among children aged five to 15 years, S. typhi was the most common in children younger than five years. The antibiotic sensitivity pattern reported in their study (from 2009 to 2011) differs substantially from what we observed as we are reporting data from 2018. They reported an incidence of MDR S. typhi ranging from 64% to 66%, 84% to 92% resistance to ofloxacin and only two cases of cefixime and CFX resistance in S. typhi isolates [15]. The age bracket in this study is similar; however, we report a tremendous fall in MDR typhoid cases and a surge in cefixime and CFX resistance. The reason behind this difference in results is likely the evolving pattern of antibiotic prescription over these years and the problems with inappropriate use of antibiotics as has been previously discussed [6,8].

In a study from Bangladesh, where the overall incidence of typhoid was 2.0 episodes/1000 person-years, in children younger than five years, it was 10.5/1000 person-years. They isolated 16 MDR S. typhi strains [16]. Additional data from India reported more than 20% of their S. typhi strains to be resistant against FQ, CFX, and azithromycin. They observed high sensitivity to first-line antityphoid agents including chloramphenicol, amoxicillin, and cotrimoxazole [9]. This is in disagreement with our results where susceptibility trends are favoring first-line agents with a high resistance towards cephalosporins and FQs observed. Another recent study from southern Pakistan reported an even higher resistance of S. typhi strains to ciprofloxacin (62.5%). CFX resistance was seen in 20.7% of strains, and 17% MDR and 3.7% XDR in typhoid cases [7]. The incidence of MDR is markedly lower in our study.

Although Pakistan has been put on warning due to its XDR typhoid outbreak, not many clinicians and microbiologists are reporting local statistics from this region. Pediatric populations, although at higher risk, have been neglected in the literature. The incidence of these superbugs in children is a public health emergency. The need to understand the importance of antibiotic preservation and monitoring enteric fever outbreak has become inevitable. Antibiotic stewardship programs are the need of the hour. Physicians and patients must practice informed and responsible use of antibiotics.

## Conclusions

Young children are vulnerable to contact typhoid infection due to improper hygiene and sanitary conditions. Resistance to second-line antityphoid agents is increasing, and there is a need to modify prescribing behavior. The outbreak of XDR typhoid seen among the children of southern Pakistan is an alarming public health concern. Policymakers, along with infectious disease specialists and public health experts, must come together to employ a multifaceted approach at the national level to tackle this superbug issue. Widespread antibiotic stewardship programs must be conducted. Water, sanitation, and hygiene conditions must be improved to eradicate typhoid.
